# Desensitization of ovalbumin-sensitized mice by repeated co-administrations of di-(2-ethylhexyl) phthalate and ovalbumin

**DOI:** 10.1186/1756-0500-2-225

**Published:** 2009-11-09

**Authors:** Søren T Larsen, Gunnar D Nielsen

**Affiliations:** 1 National Research Centre for the Working Environment, Lersø Parkallé 105, DK-2100 Copenhagen Ø, Denmark

## Abstract

**Background:**

The plasticizer di-(2-ethylhexyl) phthalate (DEHP) has been shown to stimulate a non-allergy related immune response with increased levels of IgG1 and IgG2a, but not IgE, after co-administration with the model allergen ovalbumin (OVA) in mice. In mice, decreased IgG1 and increased IgG2a have been associated with the development of mucosal tolerance towards inhaled allergens. As DEHP selectively promote formations of IgG1 and IgG2a without stimulating the IgE response, it was hypothesized that DEHP may suppress an established IgE mediated allergic response. Mice pre-sensitised to OVA were repeatedly co-exposed to DEHP and OVA and the effects were evaluated on the levels of OVA-specific antibodies, *ex vivo *cytokine levels and the degree of lung inflammation after challenge with an OVA aerosol.

**Findings:**

Compared to the OVA-sensitised control mice, multiple co-exposures to DEHP+OVA reduced the IgG1 level and reduced the IgE/IgG2a ratio. This suggests that DEHP may attenuate allergic sensitisation, as the IgE/IgG2a ratio has been shown to correlate with the degree of anaphylaxis. Nevertheless, no effect of DEHP exposures was seen on inflammatory cells in bronchoalveolar lavage fluid and on cytokine levels in spleen cell culture.

**Conclusion:**

Data from humane and murine studies suggest that DEHP may attenuate the allergic response. More studies are necessary in order to assess the size of this effect and to rule out the underlying mechanism.

## Background

The plastizicer di-(2-ethylhexyl) phthalate (DEHP) is widely distributed in the environment and DEHP is, for example, present in house dust [1], which also contains allergens e.g. from house dust mites. Therefore, the potential allergy-promoting effect of DEHP and other phthalates was evaluated in several recent studies [2]. Although some of the epidemiological studies suggested that phthalates promote allergic sensitisation [3,4], these findings could not be confirmed in controlled animal studies [5-7]. In mice, co-administration of DEHP with the model allergen ovalbumin (OVA) stimulated production of the immunoglobulins IgG1 and IgG2a but not IgE [6,7]. IgE plays a central role in many allergic diseases, whereas the role of IgG1 is less clear. IgG1 is a Th2-dependent antibody that may be anaphylactic in the mouse at high allergen exposures [8,9]. On the other hand, it has been proposed that IgG1 may constitute the murine equivalent to the human IgG4 isotype, which may protect against symptoms of allergy [10]. In mice, decreased IgG1 and increased IgG2a have been associated with the development of mucosal tolerance towards inhaled allergens [11]. If DEHP selectively promote formations of IgG1 and IgG2a without stimulating the IgE response, it could be hypothesized that DEHP may be able to suppress elicitation of an allergic response. This hypothesis is supported by a recent study showing that house dust samples spiked with DEHP (2 mg DEHP/gram dust) attenuated biomarkers of inflammation in the nasal mucosa of house dust mite allergic subjects [12]. The aim of the present study is to investigate whether repeated co-administrations of DEHP and OVA to pre-sensitized mice attenuate the allergy-related immune response. Assessments were based on the levels of OVA-specific antibodies, *ex vivo *cytokine levels, and the degree of allergic lung inflammation after challenge with an OVA aerosol.

## Methods

### Mice

Inbred female BALB/cJ mice aged 5-6 weeks were purchased from Taconic M&B, Ry, Denmark, and housed in polypropylene cages (380 × 220 × 150 mm) with pinewood sawdust bedding (Lignocel S8, Brogaarden, Denmark). The cages, each housing up to 10 mice, were furnished with bedding materials, gnaw sticks and cardboard tubes. The photo-period was from 6 a.m. to 6 p.m., and the temperature and mean relative humidity in the animal room were 19-22°C and 43 ± 8% (SD), respectively. Cages were sanitized twice weekly. Food (Altromin no. 1324, Altromin, Lage, Germany) and tap water were available *ad libitum*. Treatment of the animals followed procedures approved by The Animal Experiment Inspectorate, Denmark.

### Chemicals

DEHP (CAS 117-81-7, purity ≥98.0%) and polyethylene glycol 400 (PEG 400, Ph. Eur. Grade, CAS 25322-68-3) were from Merck, Hohenbrunn, Germany. The Al(OH)_3 _adjuvant formulation was Alhydrogel from Brenntag Biosector, Frederikssund, Denmark. Chicken egg OVA (CAS 9006-59-1) was grade V (purity ≥98%) from Sigma-Aldrich, St. Louis, MO, USA.

For use in cell culture, OVA was purified to remove endotoxins by means of an EndoTrap^® ^red kit (Profos, Regensburg, Germany), according to the operating procedures of the manufacturer. Purifying the OVA solution (10 mg/mL) reduced the endotoxin content from 25.6 IU/mg to 1.2 IU/mg, *i.e. *by more than 95%.

### Immunization procedure

**Figure 1:**
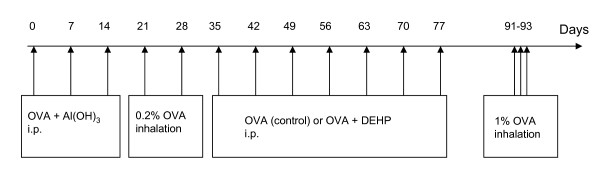
**Protocol used for sensitization/de-sensitization of animals**. Mice were sensitized by three i.p. injections of ovalbumin (OVA) and Al(OH)_3 _on day 0, 7 and 14, followed by two OVA aerosol challenges. Afterwards, the mice were treated either by i.p injections of OVA (control group) or a combination of OVA and di-(2-ethylhexyl) phthalate (DEHP) (immunotherapy group) on days 35, 42, 49, 56, 63, 70 and 77. Additionally, mice were challenged by an OVA aerosol on three consecutive days (91-93).

Mice were immunized to OVA by intraperitoneal (i.p.) injections of 1 μg OVA in combination with 270 μg Al(OH)_3 _in 100 μl 0.9% saline on day 0 (cf. Fig 1). Mice were boosted on day 7 and 14 with 0.1 μg OVA in combination with 270 μg Al(OH)_3 _in 100 μl 0.9% saline. The animals were exposed 20 min to an aerosol of 0.2% OVA on days 21 and 28 using a Pari Star nebulizer (PARI GmbH, Starnberg, Germany). This sensitization procedure was followed by seven once-weekly i.p. injections of 0.1 μg OVA alone or co-administration of 0.1 μg OVA and 100 μg DEHP (days 35, 42, 49, 56, 63, 70 and 77) in 50 μl PEG 400 vehicle (for details see [7]). Finally, mice were exposed 20 min to an aerosol of 1% OVA on three consecutive days (day 91, 92, 93). Mice were euthanized on day 94, blood was collected, broncho-alveolar lavage (BAL) was performed and the spleens were excides. BAL was performed as described previous [13]. Briefly, the total BAL cell numbers were achieved using a haemocytometer. After cytospin (1000 × g, 4 min, RT), cells were stained by May-Grünwald/Giemsa and were identified by standard morphology. Cells were differentiated into neutrophils, eosinophils, epithelial cells, lymphocytes and macrophages. For each slide, 200 cells were counted using 400 × magnifications and the percentage of each cell type was calculated.

### Ex vivo lymphocyte culture and measurement of cytokine production

On termination of the experiment, the spleens were excised and disintegrated by means of a 70 μm nylon cell strainer (BD Falcon, from BD Falcon Biosciences, Bedford, MA, USA). Cells were cultured in 24-well plates (Nunc, Denmark) with 1.0 mL cell suspension per well (2.7 × 10^6 ^cells/mL). Growth medium was RPMI 1640 (Cambrex, Belgium) supplemented with Nutridoma (1%), gentamycin (50 μg/mL), and monothioglycerol (13.5 μL/L). Cells were either unstimulated or stimulated with 1 mg/mL of LPS-purified OVA. After 5 days, supernatants were harvested and analyzed for cytokines.

Levels of IL-4, IL-5, IL-10 and IFN-γ in BAL fluids were assayed by ELISA kits (eBioscience, San Diego, USA) according to manufacturer's description.

### Antibody production

At the end of the experiment, mice were anaesthesized and blood samples were collected by heart puncture. Sera were assayed for content of OVA-specific IgE, IgG1 and IgG2a. ELISAs were performed as described previously [14].

### Statistics

Antibody levels, numbers of inflammatory cells in BAL fluid and *ex vivo *cytokine levels in the DEHP+OVA group were compared pair-wise to the OVA control group by the Mann-Whitney's *U*-test. A p-value of less than 0.05 was considered statistically significant. Calculations were performed using the Minitab Statistical Software, Release 14 Xtra (Minitab Inc., PA, USA).

## Results

### Antibody levels

**Figure 2:**
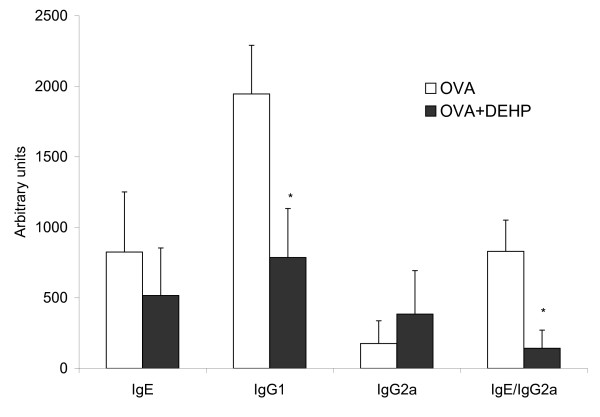
**Levels of OVA-specific antibodies**. Levels of ovalbumin (OVA)-specific IgE, IgG1 and IgG2a antibodies in serum on day 94. Levels are medians of 9-10 mice with error bars representing the 75^th ^percentiles. Statistically difference between the OVA and OVA+DEHP group is indicated by * (p < 0.05).

With the purpose to evaluate the properties of DEHP as adjuvant in immunotherapy, mice were repeatedly exposed i.p. to a combination of DEHP and OVA. The mice in the DEHP+OVA group had a significantly (p = 0.013) lower level of IgG1 than mice only exposed to OVA (cf. Fig 2). Repeated DEHP exposures tend to have a suppressive effect on IgE, although this suppression did not reach statistical significance (p = 0.076). Furthermore, there was a non-significant (p = 0.092) increase in the IgG2a level in mice repeatedly exposed to DEHP+OVA compared to the OVA control mice. The IgE/IgG2a ratio was significantly (p = 0.024) lower in the DEHP+OVA group compared to the OVA control group (Fig 2).

### Splenocyte culture cytokines

**Figure 3:**
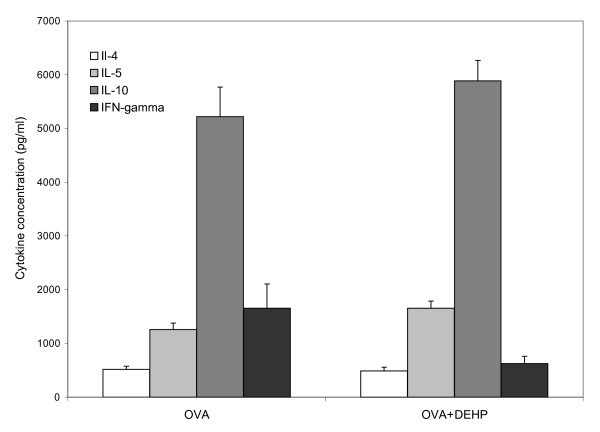
**Levels of cytokines in splenocyte culture supernatant**. For details cf. the method section. Levels are mean with SEM of 9-10 mice.

As apparent from Fig 3, there were no differences in the levels of IL-4, IL-5, IL-10 or IFNγ between the OVA and DEHP+OVA groups. In the unstimulated control cultures, levels of all cytokines were below the limit of detection.

### BAL cell

**Figure 4:**
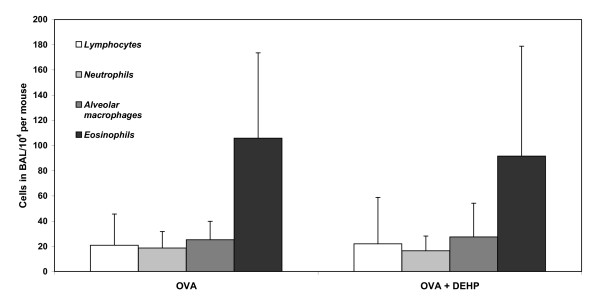
**Inflammatory cells in BAL fluid**. Inflammatory cells in broncho-alveolar lavage (BAL). Lungs were lavaged with 4 times 0.8 ml saline and cells were counted and differentiated using standard morphology. Bars indicate median values of 9-10 mice and the error bars indicate the 75^th ^percentiles.

Our protocol gave rise to a marked lung inflammation dominated by high numbers of eosinophils both in the OVA and DEHP+OVA groups as seen from Fig 4. Furthermore, a high number of neutrophils and lymphocytes were present in both groups. Pair-wise comparisons revealed that the number of neutrophils, eosinophils, epithelial cells, lymphocytes and macrophages, respectively were equal in both groups.

The epithelial cell numbers were low in both groups (data not shown).

## Discussion

This study hypothesized that DEHP can promote desensitization to IgE mediated allergy. As an industrial chemical, DEHP may have no direct potential as an adjuvant in immunotherapy. However, if DEHP can promote desensitization, this will bring additional support to results from previous studies [6,7,15,16] which showed that DEHP did not promote development of IgE mediated allergy. Also, if DEHP may promote desensitization, it may be of interest to study the adjuvant mechanisms with the purpose to develop novel adjuvants for immunotherapy of IgE mediated asthma and rhinitis. IgE mediated allergy is promoted by T-helper (Th) 2 lymphocytes producing IL-4, IL-5 and IL-13 [17,18]. Immunotherapy is used to manage IgE mediated rhinitis and asthma [17-19] by down-regulation of the Th2 response by promoting a Th1 response [19] due to production of interferon (IFN) γ, IL-2 and IL-12 [17] and up-regulation of regulatory T lymphocytes, producing IL-10 and transforming growth factor (TGF) β [17,18]. As DEHP did not alter the level of IL-10 in the present study, no conclusion can be drawn on the activity of regulatory T lymphocyte.

We established an allergic airway inflammation in mice dominated by eosinophilic inflammation and high levels of IgE and IgG1 antibodies against the model allergen OVA. Immunotherapy with multiple exposures to DEHP+OVA significantly reduced the serum level of OVA-specific IgG1 and the ratio IgE/IgG2a compared with immunotherapy with OVA alone. Furthermore, borderline to significant reduction was seen on IgE and borderline increase of IgG2a level following multiple treatments with DEHP+OVA exposure. The antibody profiles indicated that DEHP promoted a mixed Th1/Th2 immune response, whereas Al(OH)_3_, as expected, showed a pure Th2-dominated profile. This is in accordance with previous observations with immunological studies of DEHP [6]. In mice, decreased IgG1 and increased IgG2a have been interpreted as development of mucosal tolerance towards inhaled allergens [11]. In a recent study, a decreased IgE/IgG2a ratio correlated well with reduced anaphylaxis [20]; mice with a low IgE/IgG2a ratio had a lower body temperature decrease than control animals and the low IgE/IgG2a ratio protected the mice from death upon allergen challenge [20].

The change in antibody pattern observed in the present study suggests that DEHP may reduce the degree of allergic sensitization in the mice. However, no effects of DEHP were seen on the BAL cell counts, suggesting that the desensitization effect of DEHP may be relatively weak. Nevertheless, our results are in line with a recent study by Deutschle and co-workers who exposed house dust mite (HDM) allergic subjects to HDM-containing dust samples spiked with DEHP (2 mg DEHP/gram dust) [12]. DEHP reduced the levels of the inflammatory cytokines, G-CSF and IL-6, in the nasal secretions after nasal challenge with HDM, which suggests that DEHP attenuates allergic inflammation. In that study, no difference was seen on self-reported symptoms also suggesting a weak effect.

## Conclusion

The very few studies investigating the desensitisation potential of DEHP suggest that DEHP may attenuate allergic sensitization. However, further studies are needed in order to draw a firm conclusion, assessing the size of the effect, and to reveal the immunological mechanisms of the desensitization.

## List of abbreviations

DEHP: Di-(2-ethylhexyl)phthalate; ELISA: Enzyme-linked immunosorbent assay; HDM: House dust mite; IL: Interleukin; LPS: Lipopolysaccaride; OVA: Ovalbumin; PEG: Polyethylene glycol; RT: room temperature; Th: T-helper cell.

## Competing interests

The authors declare that they have no competing interests.

## Authors' contributions

Both authors designed the study. STL was responsible for the laboratory experiments. Both authors contributed to data analyses and preparation of manuscript.
